# Antisense Activity across the *Nesp* Promoter is Required for *Nespas*-Mediated Silencing in the Imprinted *Gnas* Cluster

**DOI:** 10.3390/ncrna1030246

**Published:** 2015-11-30

**Authors:** Charlotte J. Tibbit, Christine M. Williamson, Stuti Mehta, Simon T. Ball, Mita Chotalia, Wade T. Nottingham, Sally A. Eaton, Mohamed M. Quwailid, Lydia Teboul, Gavin Kelsey, Jo Peters

**Affiliations:** 1 MRC Harwell, Mammalian Genetics Unit, Harwell Campus, Oxfordshire OX110RD, UK; E-Mails: m.quwailid@har.mrc.ac.uk (M.M.Q.); charlotte.tibbit@dpag.ox.ac.uk (C.J.T.); c.williamson@har.mrc.ac.uk (C.M.W.); stuti.mehta@wolfson.oxon.org (S.M.); s.ball@har.mrc.ac.uk (S.T.B.); wadenottingham@google.com (W.T.N.); S.Eaton@victorchang.edu.au (S.A.E.); 2 Epigenetics Programme, The Babraham Institute, Cambridge CB223AT, UK; E-Mails: gavin.kelsey@babraham.ac.uk (G.K.); mita.chotalia@googlemail.com (M.C.); 3 Mary Lyon Centre, MRC Harwell, Harwell Campus, Oxfordshire OX110RD, UK; E-Mail: l.teboul@har.mrc.ac.uk; 4 Centre for Trophoblast Research, University of Cambridge, Cambridge CB23EG, UK; 5 Current address: MRC Functional Genomics Unit, Department of Physiology Anatomy & Genetics, Le Gros Clark Building, University of Oxford, South Parks Road, Oxford OX13QX, UK; 6 Current Address: MRC Harwell, Harwell Campus, Oxfordshire OX110RD, UK; 7 Current address: GI Division, Their 340, Massachusetts General Hospital, Harvard Medical School, Boston, MA 02114, USA; 8 Current address: Mary Lyon Centre, MRC Harwell, Harwell Campus, Oxfordshire OX110RD, UK; 9 Current address: Genome Function Group, MRC Clinical Sciences Centre, Imperial College London, Hammersmith Hospital Campus, London W120NN, UK; 10 Current address: West London Free School, 2 Bridge Avenue, Hammersmith, London W69JP, UK; 11 Current address: Molecular, Structural and Computational Biology Division, Victor Chang Cardiac Research Institute, Darlinghurst, NSW 2010, Australia

**Keywords:** long non-coding RNA, antisense, genomic imprinting, epigenetic silencing, *Nespas*, *Nesp*, *Gnas* cluster

## Abstract

Macro long non-coding RNAs (lncRNAs) play major roles in gene silencing in inprinted gene clusters. Within the imprinted *Gnas* cluster, the paternally expressed *Nespas* lncRNA downregulates its sense counterpart *Nesp*. To explore the mechanism of action of *Nespas*, we generated two new knock-in alleles to truncate *Nespas* upstream and downstream of the *Nesp* promoter. We show that *Nespas* is essential for methylation of the *Nesp* differentially methylated region (DMR), but higher levels of *Nespas* are required for methylation than are needed for downregulation of *Nesp*. Although *Nespas* is transcribed for over 27 kb, only *Nespas* transcript/transcription across a 2.6 kb region that includes the *Nesp* promoter is necessary for methylation of the *Nesp* DMR. In both mutants, the levels of *Nespas* were extraordinarily high, due at least in part to increased stability, an effect not seen with other imprinted lncRNAs. However, even when levels were greatly raised, *Nespas* remained exclusively *cis*-acting. We propose *Nespas* regulates *Nesp* methylation and expression to ensure appropriate levels of expression of the protein coding transcripts *Gnasxl* and *Gnas* on the paternal chromosome. Thus, *Nespas* mediates paternal gene expression over the entire *Gnas* cluster via a single gene, *Nesp*.

## 1. Introduction

Imprinted genes generally occur in clusters and most imprinted clusters contain at least one long non-coding RNA [1]. Parental specific expression of genes within a cluster is under the overall control of an imprinting control region (ICR). For four well characterised clusters, the ICR contains the promoter of an unusually long non-coding RNA (lncRNA) that is exclusively expressed from the paternal allele and runs antisense to at least one protein coding gene within the cluster. All four lncRNAs, *Airn* in the *Igf2r* cluster [2], *Kcnq1ot1* in the *Kcnq1* cluster [3], *Nespas* in the *Gnas* cluster [4] and *Ube3aas* in the *Snrpn* cluster [5,6] are required for silencing one or more protein coding genes within their respective clusters.

**Figure 1:**
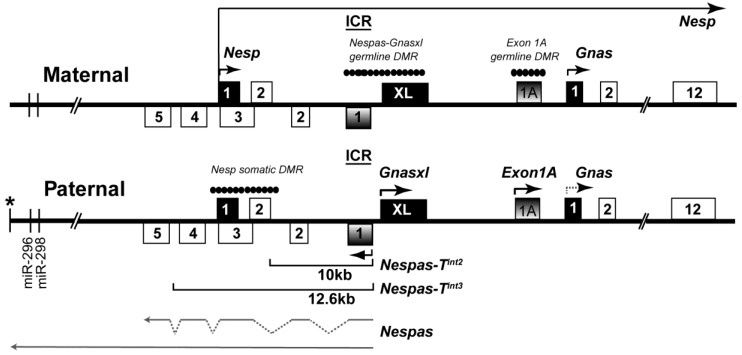
Organisation of the *Gnas* cluster. First exons of protein coding transcripts are shown as filled boxes and first exons of noncoding transcripts as shaded boxes. Arrows show the initiation and direction of transcription. *Nesp* is transcribed across the cluster. The arrow corresponding to the paternal *Gnas* allele is shown as a dotted line to indicate that the paternal *Gnas* allele is repressed tissue specifically. The 10 kb and 12.6 kb lines represent the new truncation mutations *Nespas-T^int2^* and *Nespas-T^int3^*. *Nespas* non-coding transcripts are shown as grey lines. * represents the termination of *Nespas* transcription, approximately 30 kb from the *Nespas* promoter. Paternally expressed microRNAs are shown as vertical lines. Filled circles represent methylated regions. ICR represents the imprinting control region.

*Gnas* is a complex imprinted cluster with three protein coding genes, *Nesp* that encodes the neurosecretory protein NESP55, *Gnas* that encodes the stimulatory G-protein Gsα and *Gnasxl* that encodes extra-large forms of Gsα [7] (Figure 1). Appropriate levels of both *Gnasxl* and *Gnas* are required for normal growth and survival [8,9,10]. The ICR for the cluster is unmethylated and active on the paternally derived allele and contains the promoter for *Nespas* [11]. *Nespas* is paternally expressed and runs antisense to its maternally expressed protein coding sense counterpart *Nesp*. Both a macro form and several spliced forms up to 1.4 kb in size of *Nespas* RNA are known [12]. *Nespas* is transcribed through the promoter of *Nesp*, which lies within a somatic differentially methylated region (DMR), that acquires methylation on the paternal allele post fertilisation [13,14,15]. When *Nespas* is not expressed the *Nesp*, DMR remains unmethylated and *Nesp* is expressed on the paternal allele, therefore the primary role of *Nespas* may be to repress *Nesp* [11].Low levels of *Nespas* expression can downregulate *Nesp* on the paternal allele through chromatin modification in the absence of DNA methylation at the *Nesp* DMR [4]. It has been proposed that DNA methylation of the *Nesp* DMR is essential to stabilise silencing of *Nesp* long term [4].

We have carried out the first study examining the effects of truncating an imprinted lncRNA to different lengths in an *in vivo* mouse model. Using two new truncation alleles, we show that expression of *Nespas* is required for methylation of the *Nesp* DMR. Our results show that a requirement for *Nespas*-mediated methylation and silencing *in cis* is that *Nespas* transcript/transcription needs to span the *Nesp* promoter. Although *Nespas* is transcribed for more than 27 kb [16,17], it is only *Nespas* transcript/transcription across a 2.6 kb region that includes the *Nesp* promoter that is required for methylation of the *Nesp* DMR. In both mutants, truncation of *Nespas* resulted in very highly elevated levels of truncated *Nespas* transcript, an effect not seen with truncation of other non-coding RNAs. However, even when *Nespas* levels were greatly raised *Nespas* remained exclusively *cis*-acting.

## 2. Results

### 2.1. *Nespas* Transcription Extends over 30 kb

Previous work had shown evidence of *Nespas* transcription approximately 30 kb downstream from its promoter [16,17]. In the present study, using 3′ RACE, we identified a poly(A) addition site, approximately 30 kb from the *Nespas* promoter and just downstream of a poly(A) signal AAUAAA, at nucleotide 122,587 (BAC sequence AL593857.10). This site represents the end of *Nespas* transcription as it aligns with the end of transcription in 12.5 dpc placenta as determined by an RNA expression tiling array analysis using 50 bp oligos spaced at 50 bp intervals [18].

### 2.2. The *Nesp* DMR Is Unmethylated When *Nespas* Is Truncated before the *Nesp* Promoter

**Figure 2:**
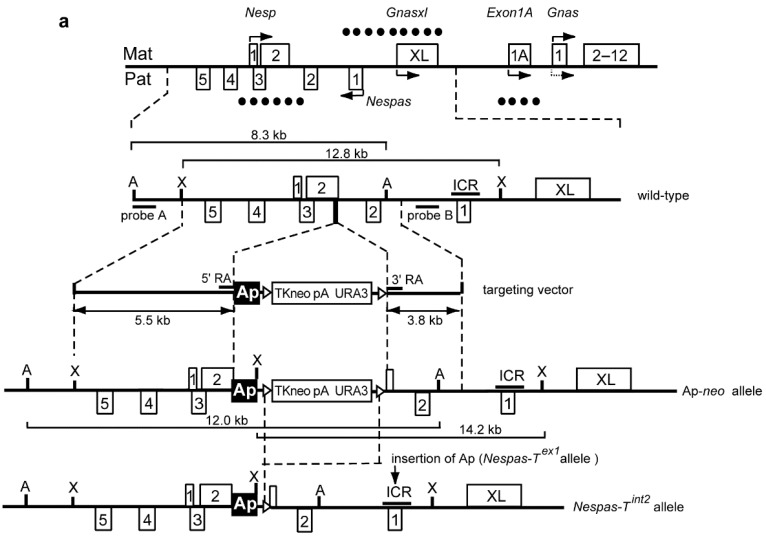

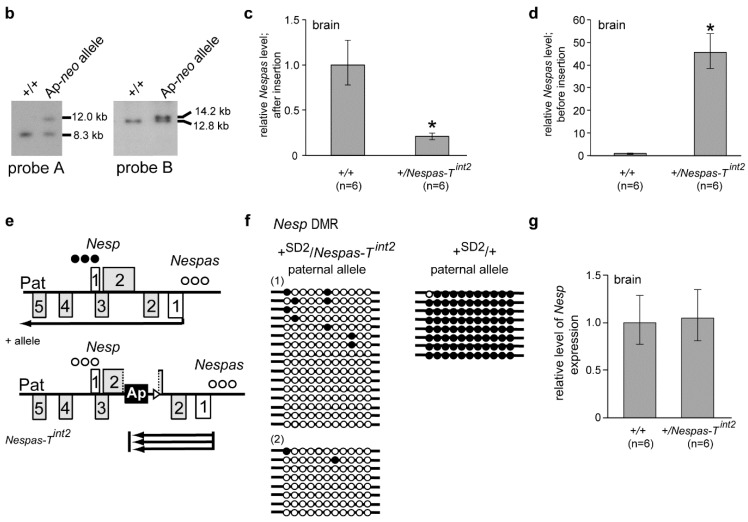
Truncation of *Nespas* before the *Nesp* promoter in *Nespas* intron 2/*Nesp* exon 2. (**a**) Schematic of the mouse *Gnas* locus showing the site of insertion of the rabbit β-globin polyadenylation cassette [2]. The targeting vector shows the location of a 1.2 kb fragment (black box labelled Ap) from the rabbit β-globin gene to truncate *Nespas* and the position of the selection cassette (TKneopA URA3), flanked by *loxP* sites (open triangles). The targeted allele was designated *Nespas-T^int2^* allele after Cre-mediated excision of the selectable marker cassette. Methylated DMRs are shown by filled circles. A, *Afl*II; X, *Xho*I; ICR, Imprinting Control Region. The arrow shows the approximate position of the polyadenylation cassette (pA) to truncate *Nespas* in exon 1 in *Nespas-T^ex1^* [4]; (**b**) Southern analysis of ES cell DNA showing correct targeting in the Ap-*neo* allele. Ap-*neo* targeted clones were identified by the presence of a 12.0 kb *Afl*II fragment detected with the 5' external probe, probe A. Correct targeting at the 3' end was confirmed by the detection of a 14.2 kb *Xho*I fragment with Probe B; (**c**) *Nespas* is downregulated after the insertion; (**d**) *Nespas* is upregulated before the insertion; (**e**) *Nespas* is truncated and abundant in +/*Nespas-T^int2^*; (**f**) Bisulphite analysis showing the *Nesp* DMR is unmethylated on the paternal allele in +^SD2^/*Nespas-T^int2^*. All clones from one individual are grouped into a block and two +^SD2^/*Nespas-T^int2^* individuals, (1) and (2), and one +^SD2^/+ individual were analysed. Each row of circles represents a clone derived from the paternal allele and each circle corresponds to a separate CpG (filled circle, methylated CpG; unfilled circle, unmethylated CpG) for nucleotides 140,450–140,800; (**g**) The *Nesp* level is unaffected in +/*Nespas-T^int2^*.

To create the truncation mutant, *Nespas-T^int2^* a polyadenylation cassette (pA) from the rabbit β- globin gene was inserted into *Nespas* intron 2 in an orientation (Ap) to truncate *Nespas* 10 kb from its promoter and downstream of the *Nesp* promoter (Figure 2a,b). Following paternal transmission, we determined whether *Nespas* was truncated by Taqman reverse transcription quantitative real-time PCR (RT-qPCR) analysis. An assay 3′ of the insertion with respect to *Nespas* transcription, specific to *Nespas* intron 4 and upstream of the *Nesp* promoter, detected an 80% reduction in *Nespas* level in +/*Nespas-T^int2^* (maternal allele precedes the paternal allele) relative to wild-type (*n* = 6, *P* = 1.0 × 10^−3^; Figure 2c). In contrast, an assay 5′ of the insertion with respect to *Nespas* transcription, specific for *Nespas* exon 1 spliced onto exon 2 and downstream of the *Nesp* promoter, detected an unexpectedly high 46 fold increase in *Nespas* in +/*Nespas-T^int2^* compared with the level in wild-type (*n* = 6, *P* = 1.5 × 10^−10^; Figure 2d). Thus, the low level of *Nespas* after the insertion compared with the high level before the insertion showed that most of the transcripts were truncated (as shown in the schematic in Figure 2e).

We next investigated the methylation status of the paternal somatic DMR at *Nesp*. Bisulphite analysis was done on brain DNA from newborn offspring arising from crosses of *Nespas-T^int2^* carrier males with SD2 females carrying the *Gnas* imprinted region from *Mus spretus*. The presence of single nucleotide variants in the parents enabled the maternal and paternal alleles of *Nesp* to be distinguished. Figure 2f shows that the paternal *Nesp* DMR was unmethylated in +/*Nespas-T^int2^*. A similar result was observed in +/*Nespas-T^int2^* mice derived from a second independently targeted clone. Thus, the normally methylated paternal *Nesp* allele was unmethylated when *Nespas* was truncated before the *Nesp* DMR in +/*Nespas-T^int2^* (Figure 2e).

To investigate expression of *Nesp* from the mutant unmethylated paternal *Nespas-T^int2^* allele, we measured transcript levels in newborn brain using a RT-qPCR Taqman assay specific for exon 1 spliced onto exon 2 of *Nesp* [4]. There was no significant difference in the level of *Nesp* transcript in newborn brain between wild-type and +/*Nespas-T^int2^* (Figure 2g), so we can conclude that there was very little, if any expression from the mutant paternal allele.

### 2.3. Lack of Methylation at the *Nesp* DMR Is Due to Truncation of *Nespas*

**Figure 3:**
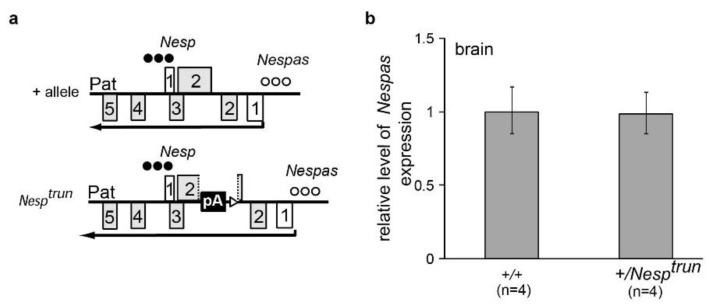

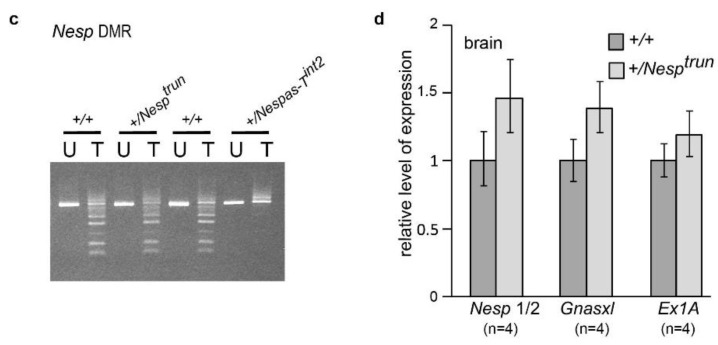
Transcription and methylation status of the *Gnas* cluster is unaltered on paternal inheritance of *Nesp^trun^*. (**a**) Schematic summary of the transcriptional and methylation status of *Nesp* and *Nespas* on the paternal allele in wild-type and +/*Nesp^trun^*. The pA insertion will truncate *Nesp* if expressed. Row of filled circles, methylated allele; row of unfilled circles, unmethylated allele; (**b**) The level of *Nespas* exon 1 spliced onto exon 2 is unaltered in +/*Nesp^trun^*; (**c**) Methylation at the *Nesp* DMR is unaffected. PCR products from bisulphite-treated DNAs were separated on 3% agarose gels as either undigested (U) or digested with *Taq*1 (T). Digestion products in the T lanes represent methylation of the CpG dinucleotide in the *Taq*1 recognition sequence. Genomic DNA from +/*Nespas-T^int2^* in which both alleles were unmethylated was included as a negative control; (**d**) The levels of *Nesp*, *Gnasxl* and *Exon1A* (*Ex1A*) are unaffected.

To determine whether the lack of methylation at the *Nesp* DMR in +/*Nespas-T^int2^* was due to truncation of *Nespas* or due to the physical disruption of the *Nesp* locus, we analysed +/*Nesp^trun^* mutant mice [19] which had the polyadenylation cassette inserted at the same location as in *Nespas-T^int2^*, but in the opposite orientation so that truncation of *Nespas* should not occur (Figure 3a). The *Nesp^trun^* allele was originally generated to truncate maternal *Nesp* as a functional test for the role of *Nesp* transcription [19], but the level of *Nespas* and the methylation status at the *Nesp* DMR on paternal inheritance were not analysed.

Here, we show that, on paternal inheritance, the insertion in *Nesp^trun^* had no effect on the level of *Nespas* (Figure 3b) and that full expression of *Nespas* from the paternal allele was associated with methylation of the *Nesp* DMR *in cis* (Figure 3c). As expected from the methylation analysis, there was no significant difference in the levels of *Nesp*, *Gnasxl*, or *Exon1A* in wild-type and +/*Nesp^trun^* (Figure 3d). Thus, our data indicate that the lack of methylation at the *Nesp* DMR in +/*Nespas-T^int2^* is due to truncation of *Nespas* rather than physical disruption of the locus. Thus, methylation of *Nesp* on the paternal allele is controlled by the *Nespas* non-coding macro RNA gene that is transcribed from a methylation sensitive promoter within the ICR of the *Gnas* cluster.

### 2.4. Truncation of *Nespas* after the *Nesp* Promoter

**Figure 4:**
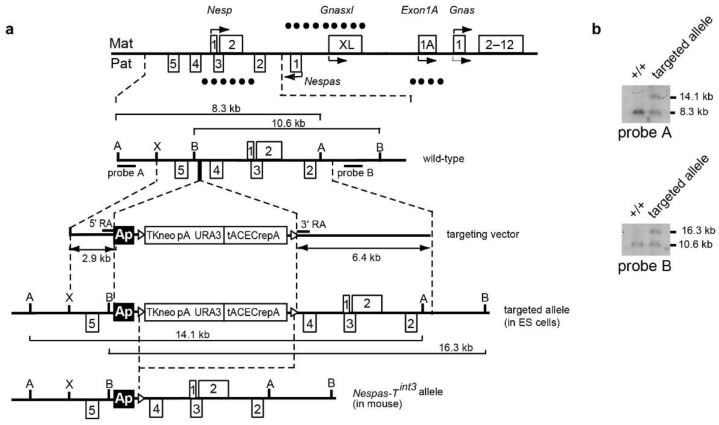

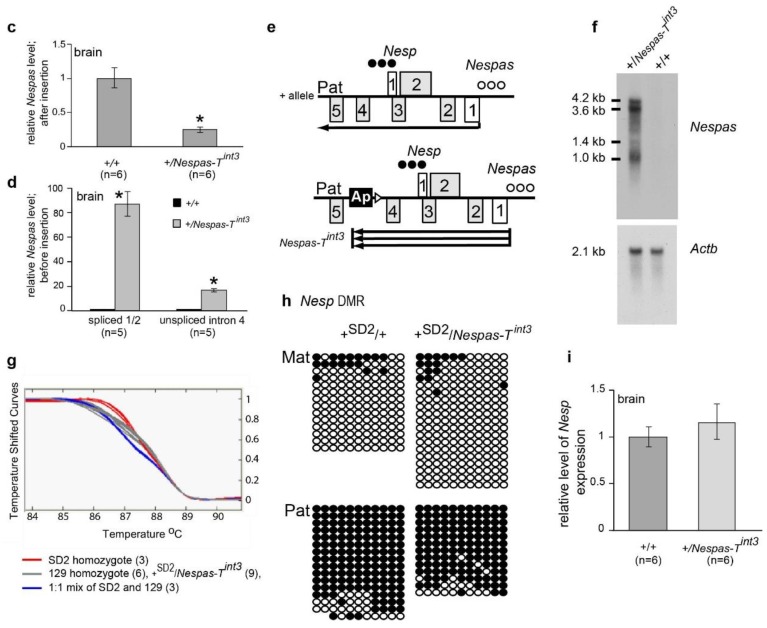
Truncation of *Nespas* after the *Nesp* promoter in *Nespas* intron 4. (**a**) Schematic of the mouse *Gnas* locus showing the site of insertion of the rabbit β-globin polyadenylation cassette (black box labelled Ap) to truncate *Nespas* and the position of the selection cassette flanked by *loxP* sites (open triangles). The selection cassette was deleted on germline transmission and the targeted allele was designated *Nespas-T^int3^*. A, *Afl*II; B, *Bsr*GI; X, *Xho*I; (**b**) Southern analysis of ES cell DNA showing correct targeting. Correctly targeted clones were identified by the presence of a 14.1 kb *Afl*II fragment detected with the 5′ external probe, probe A. Correct targeting at the 3′ end was confirmed by the detection of a 16.3 kb *Bsr*GI fragment with probe B; (**c**) *Nespas* is downregulated after the insertion; (**d**) *Nespas* is upregulated before the insertion. There was a greater fold difference in the spliced form (exon 1 spliced onto exon 2) than the unspliced form (intron 4); (**e**) *Nespas* is truncated and abundant in +/*Nespas-T^int3^*; (**f**) Northern blot assay of *Nespas* and loading control *Actb*; (**g**) Light Scanner melt profile, using a SNP in *Nespas* exon 3, to show *Nespas* is expressed from the paternal allele in +/*Nespas-T^int3^*; (**h**) Bisulphite analysis showing the *Nesp* DMR is unmethylated on the maternal allele and methylated on the paternal allele in +^SD2^/*NespasT^int3^*; (**i**) The *Nesp* level is unaffected in +/*Nespas-T^int3^*.

To narrow down the region of *Nespas* responsible for silencing, we inserted the polyadenylation cassette from the rabbit β-globin gene into *Nespas* intron 4 in an orientation (Ap) to truncate *Nespas* 12.6 kb from its promoter and upstream of the *Nesp* promoter (nucleotides 139,675–140,530; defined in Figure S1). The gene targeting details are shown in Figure 4a,b, and we designated the new allele *Nespas-T^int3^*.

The mutant allele *Nespas-T^int3^* should give rise to a *Nespas* transcript that spans the *Nesp* promoter. Following paternal transmission we determined whether *Nespas* was truncated by Taqman RT-qPCR analysis. An assay immediately 3′ of the insertion with respect to *Nespas* transcription, specific to *Nespas* intron 4 and upstream of the *Nesp* promoter, detected a 75% reduction in *Nespas* level in +/*Nespas-T^int3^* relative to wild-type (*n* = 6; *P* = 8.2 × 10^−6^; Figure 4c). In contrast, an assay 5′ of the insertion with respect to *Nespas* transcription, specific for *Nespas* exon 1 spliced onto exon 2 showed an 87-fold increase in *Nespas* compared to wild-type littermates in +/*Nespas-T^int3^* on a 129S9/SvEvH background generated from one clone (*n* = 5, *P* = 2.1 × 10^−11^; Figure 4d), a 59-fold increase in +/*Nespas-T^int3^* generated from a second independently targeted clone (*n* = 6, *P* = 3.0 × 10^−4^) and an 80-fold increase in +/*Nespas-T^int3^* on a SD2 background (*n* = 6; *P* = 5.9 × 10^−11^). Thus, the increase in *Nespas* in two independently targeted clones suggests the effect was due to the insertion rather than a non-specific abnormality in the ES cells. Furthermore, as *Nespas* was truncated upstream of *Nesp* in +/*Nespas-T^int3^*, an RT-qPCR assay specific for the truncated unspliced form of *Nespas* could be carried out. An RT-qPCR Taqman assay in *Nespas* intron 4, 5' of the insertion with respect to *Nespas* transcription, and upstream of the *Nesp* promoter (Table S2) showed that the unspliced form of *Nespas* was raised 17-fold when compared with the wild-type level (*n* = 5; *P* = 5.5 × 10^−8^; Figure 4d). Once again, the low level of *Nespas* detected after the insertion compared with the high level detected before the insertion showed that most of the transcripts were truncated as summarised in the schematic in Figure 4e.

The raised expression of *Nespas* in +/*Nespas-T^int3^* was verified by RNA blot analysis using a single stranded probe that would detect spliced and unspliced transcripts. The *Nespas* transcripts which are detected as a smear [20], were highly elevated in newborn brain from +/*Nespas-T^int3^* compared to wild-type (Figure 4f).

Furthermore, *Nespas* was shown to be monoallelically expressed from the paternal allele by melt curve analysis (Figure 4g). The melt curves show a decrease in fluorescence as the duplexes melt and the bound LCGreen plus is released into solution. The melt curves for the heteroduplex control (1:1 mix of 129S9/SvEvH and SD2 cDNA; blue line) and for the homoduplex controls 129S9/SvEvH and SD2 (grey line and red line, respectively) were distinct, thus making it possible to distinguish allele-specific *Nespas* expression. The melt curve for the duplex derived from +/*Nespas-T^int3^* brain (grey) aligned with the curve for the homoduplex control 129S9/SvEvH which was also grey. Thus, *Nespas* is expressed from the 129S9/SvEvH allele in +/*Nespas-T^int3^*, which is the targeted paternally inherited allele.

### 2.5. The Paternal *Nesp* DMR Is Methylated When *Nespas* Is Truncated after the *Nesp* Promoter

We next investigated the effect of a high level of *Nespas* RNA truncated after the *Nesp* promoter on the methylation of the *Nesp* DMR on the paternal allele in +/*Nespas-T^int3^*. Bisulphite analysis of both the maternal and paternal alleles of one wild-type (+^SD2^/+) and two mutant (+^SD2^/*Nespas-T^int3^*) newborn brains showed no loss of methylation on the paternal allele of +/*Nespas-T^int3^* compared with that of a wild-type sibling and there was no gain of methylation on the maternal allele (Figure 4h). As expected from the methylation analyses, an RT-qPCR Taqman assay showed there was no difference in the level of *Nesp* in +/*Nespas-T^int3^* when compared to the wild-type level (Figure 4i) indicating that *Nesp* was not expressed from the mutant paternal allele. Furthermore, there was no difference in *Nesp* level in mice generated from a second independently targeted clone.

Thus, paternally expressed *Nespas* must span the *Nesp* promoter in order to methylate the paternal *Nesp* DMR. Interestingly, even though *Nespas* was very abundant in +/*Nespas-T^int3^* the *Nesp* DMR on the maternal allele remained unmethylated, thus showing that increased *Nespas* does not force the silencing of *Nesp in trans*.

### 2.6. Stability of Truncated *Nespas* RNA in +/*Nespas*-T^int3^

The increased abundance of *Nespas* RNA in +/*Nespas-T^int2^* and +/*Nespas-T^int3^* compared to wild-type could be due to greater stability of truncated *Nespas* RNA. The stability of *Nespas* RNA was analysed in +/*Nespas-T^int3^* and wildtype sibs by inhibiting total cellular transcription in mouse embryonic fibroblast (MEF) cells using Actinomycin D, a procedure used previously to test the stability of *Airn* RNA [21].

**Figure 5:**
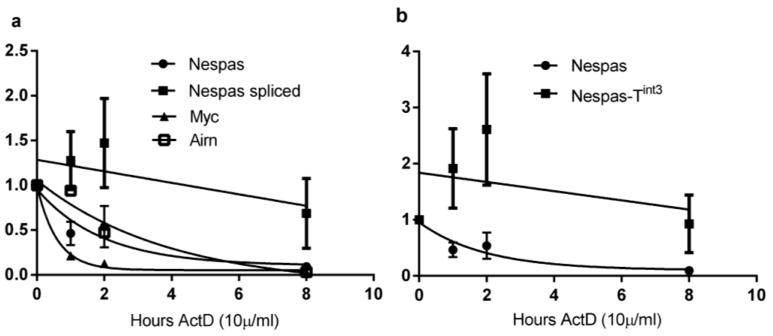
Stability of *Nespas* RNA (**a**) Wild-type *Nespas* RNA. Mouse embryonic fibroblasts MEFs were treated with ActinomycinD (10 μg/mL). Control untreated samples were set to 100% and Actinomycin D treated samples are shown as a % of controls. A one-phase exponential decay curve was constructed by Prism4 for unspliced *Nespas* RNA (black circle), spliced *Nespas* RNA (black square), *Myc* RNA (black triangle) and *Airn* RNA (open square); (**b**) Stability of *Nespas-T^int3^* RNA. Wild-type *Nespas* RNA (black circle) and *Nespas-T^int3^* RNA (black square).

*Myc* and *Airn* were used as controls for unstable RNAs (Figure 5a). Most *Myc* RNA had disappeared after one hour of exposure, and most *Airn* and unspliced *Nespas* RNAs after eight hours exposure, to Actinomycin D. The half-life of *Myc* RNA was 0.4 h, that of *Airn* RNA was 2.1 h, identical to that previously found in MEFs [21]. Unspliced *Nespas* RNA was also found to be unstable with a half-life of 1.6 h. The spliced forms showed increased stability with almost 70% still present after eight hours exposure (Figure 5a), and we were unable to obtain a half-life for the spliced forms under the experimental conditions used. The mutant truncated unspliced *Nespas* RNA in +/*Nespas-T^int3^* with a half-life of 18.6 h was over 10 times as stable as wild-type (Figure 5b).

### 2.7. Reduced Level of Gnasxl on Paternal Transmission of *Nespas*-T^int2^

**Figure 6:**
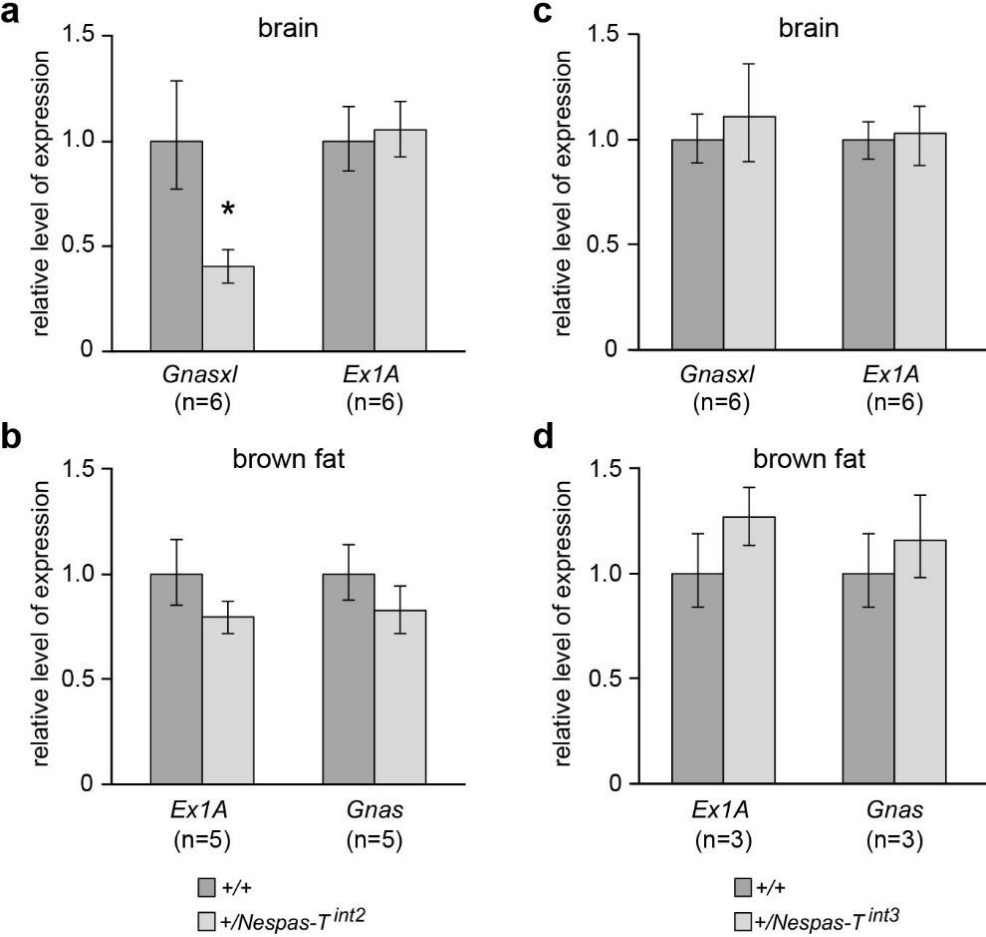
Levels of *Gnasxl*, *Exon1A* and *Gnas* in *+*/*Nespas-T^int2^* and +/*Nespas-T^int3^*. (**a**) and (**b**) *Gnasxl* is downregulated in +/*Nespas-T^int2^* whereas *Exon1A* (*Ex1A*) and *Gnas* are unaffected; (**c**) and (**d**) *Gnasxl*, *Exon1A* and *Gnas* are unaffected in +/*Nespas-T^int3^*. *Gnasxl* and *Ex1A* levels were measured in newborn brain whereas *Gnas* and *Ex1A* were analysed in brown fat, a tissue in which *Gnas* is predominantly expressed from the maternal allele [25,26].

The level of *Gnasxl* was reduced by 60% in +/*Nespas-T^int2^* when compared with wild-type (*n* = 6, *P* = 3.8 × 10^−4^, Figure 6a). Although +/*Nespas-T^int2^* appeared to be normal at birth, were generated at the expected Mendelian frequency (49.6% of 244 newborns) and showed normal viability thereafter, within a few days they were seen to be thinner than their wild type litter mates and by postnatal day 14 weighed 67.3% of their wild-type litter mates (*P* = 8.35 × 10^−11^; +/+ *n* = 27, +/*Nespas-T^int2^ n* = 23). Growth retardation is typical of mutants with reduction in *Gnasxl* levels [8,15,22,23,24]. Expression of *Exon1A* and *Gnas* was found to be unaffected (Figure 6a,b).

+/*Nespas-T^int3^* mice were generated at the expected Mendelian frequency at birth (45.6% of 171 newborns) and showed normal viability thereafter. No adverse phenotype was observed which is in keeping with the normal expression of *Gnasxl, Exon1A,* and *Gnas* found in these mice (Figure 6c,d).

## 3. Discussion

From previous work, it was known that methylation of the *Nesp* DMR correlates with silencing of *Nesp* expression [4,11]. We have shown here that methylation of the *Nesp* DMR is dependent on transcription of *Nespas* across a 2.6 kb region that includes the *Nesp* promoter. In the present study, we see truncation of most but not all *Nespas* transcripts in *Nespas*-*T^int2^*; around 20% cross the *Nesp* DMR, but this weak *Nespas* expression was insufficient for methylation of the DMR. This result extends earlier findings that the DMR was unmethylated when *Nespas* was very weakly expressed [4]. Thus, *Nespas* resembles *Airn*, [2] the well studied antisense long non-coding RNA at the *Igf2r* cluster, in that both run through, and are required for methylation of the promoters of their respective sense counterparts, *Nesp* and *Igf2r*. Furthermore, when *Nespas* and *Airn* [27] are weakly expressed *Nesp* and *Igf2r* do not acquire methylation.

Methylation of the *Nesp* DMR occurs between 3.5 dpc and 8.5 dpc [15,28]. *Nesp* is not robustly expressed until 6.5 dpc [24,29], but *Nespas* is expressed much earlier, from the two cell stage onwards [29]. Expression of *Nespas* is associated with elevated levels of histones H3K36me3 and unmethylated H3K4 at the *Nesp* promoter [4]. H3K36me3 and unmethylated H3K4 are permissive for DNA methylation [30,31,32], and so it is feasible that transcription of *Nespas* through the *Nesp* promoter region enables DNA methylation through the deposition of histone marks permissive for DNA methylation. Whether DNA methylation of the *Nesp* promoter is instrumental in initiating silencing of *Nesp* is not known and DNA methylation may be required to stabilise silencing of *Nesp* once silencing is underway.

Methylation of the *Nesp* DMR may also be required to maintain full expression of *Gnasxl*. *Nespas-T^int2^* is the fourth mutant in which the *Nesp* DMR is unmethylated on paternal inheritance and *Gnasxl* is downregulated. Unlike *Nespas-T^int2^*, there is expression of *Nesp* from the mutant paternal allele in the three other mutants, *ΔNAS-DMR*, *Nespas-T^ex1^* and *Nesp-T^int^* [4,11,24]. Furthermore, *Gnasxl* expression is reduced even though the *Gnasxl* DMR is unmethylated [4,11]. These findings are consistent with presence of a silencer for *Gnasxl* within the *Nesp* DMR that is active when the *Nesp* DMR is unmethylated and inactive when the *Nesp* DMR is methylated. Deletion of the paternal methylated *Nesp* DMR does not affect expression of *Gnasxl* [25] which accords with the prediction of a silencer. We propose that DNA methylation at the *Nesp* DMR is needed to inactivate a silencer of *Gnasxl*.

Although the paternal *Nesp* DMR was unmethylated in *Nespas*-*T^int2^*, the expression of *Nesp* was not detectably affected and *Nesp* remained silent. It was known that very low levels of *Nespas* can downregulate *Nesp* [4] and therefore in *Nespas***-***T^int2^* it is likely that the 20% or so of wild-type *Nespas* transcripts that are not truncated and cross the *Nesp* DMR are sufficient to silence *Nesp*. However, we cannot exclude the possibility that transcription of *Nesp* in *Nespas*-*T^int2^* was diminished by the close proximity of the insertion to the *Nesp* promoter. Taken together with findings that total loss of *Nespas* expression across the *Nesp* promoter results in complete de-repression of paternal *Nesp* [4,11] it can be concluded that expression of *Nespas* is definitely required for silencing of *Nesp*. Thus, although silencing the *Nesp* promoter only requires weak expression of *Nespas*, higher levels of *Nespas* are required for DNA methylation of the silenced region.

It is not known whether it is transcription of *Nespas* or the *Nespas* transcript that is instrumental is silencing *Nesp*. Given the sense-antisense overlap *Nespas* may downregulate *Nesp* by a transcription based mechanism as reported for the silencing of *Igf2r* by *Airn* [33], or by a transcript based RNA interference mechanism. Even when transcript levels were highly elevated *Nespas* acted exclusively in *cis*, hinting that transcription of *Nespas* through the *Nesp* promoter rather than the *Nespas* transcript itself may result in silencing of *Nesp*. However, it has been reported that *Nespas* RNA is associated with the chromatin modifying Polycomb repressive complex 2, so a silencing role for the *Nespas* transcript cannot be excluded [34].

We show here that wild-type unspliced *Nespas* ncRNA is unstable, with the lack of stability resembling that found for *Airn* and *Tsix* ncRNAs [21,35]. As found with *Airn*, the spliced forms of *Nespas* are more stable than the unspliced form which may indicate that unspliced *Nespas* RNA is rapidly degraded. Unlike the long non coding antisense *Airn* and *Kcnq1ot1* RNAs whose expression levels are unaltered when truncated [2,3,33], truncation of *Nespas* RNA results in very high increase in expression levels. Truncated *Nespas* RNA is much more stable than the wild-type form, and this increased stability may well account in large part for the increased expression levels of truncated *Nespas*.

**Figure 7:**
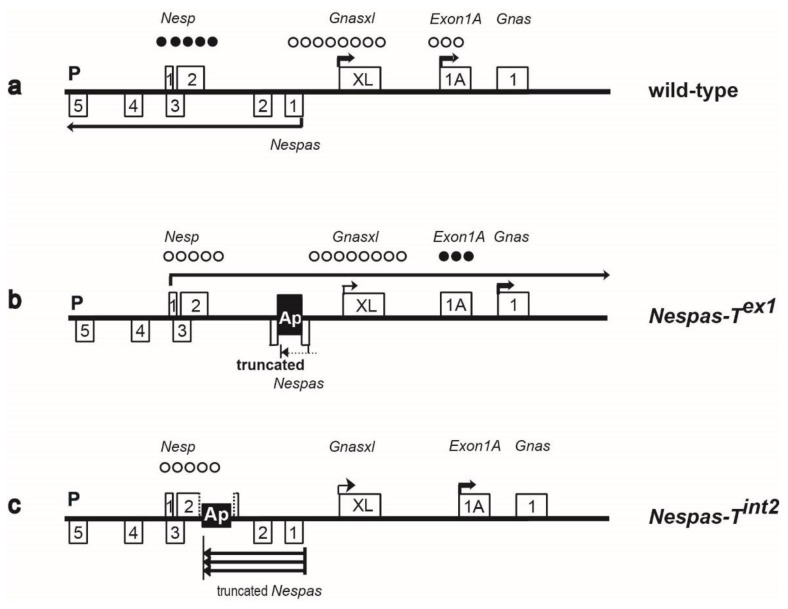

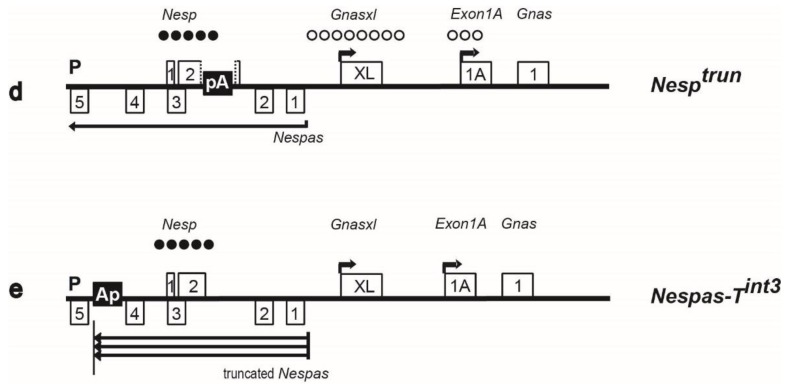
Summary of the transcriptional and methylation status of the paternal allele of the imprinted *Gnas* cluster in wild-type and mutant mice. (**a**) wild-type; (**b**) +/*Nespas-T^ex1^*; (**c**) +/*Nespas-T^int2^*; (**d**) +/*Nesp^trun^*; (**e**) +/*Nespas-T^int3^*. The Ap insertion truncates *Nespas* and the pA insertion will truncate *Nesp* if expressed. The approximate positions of the differentially methylated regions (DMRs) are shown by rows of filled circles on the methylated allele and unfilled circles on the unmethylated allele. Arrows show the start and direction of transcription, with thin arrows indicating weak transcription. Multiple arrows in (**c**) and (**e**) show increased levels of truncated *Nespas*. Exons 2–12 of *Gnas* are not shown. The figure is not drawn to scale. Information for (**b**), +/*Nespas-T^ex1^*, taken from [4,24].

There is mounting evidence that the role of *Nespas* is to suppress expression of *Nesp* and induce methylation of the *Nesp* DMR on the paternal chromosome, thereby indirectly controlling paternal expression of protein coding transcripts *Gnasxl* and *Gnas*. On the paternal chromosome in wild-type, *Nesp* is repressed, the *Nesp* DMR is methylated, *Gnasxl* is fully expressed and *Gnas* is suppressed tissue specifically (Figure 1 and Figure 7). However, whereas methylation of the *Nesp* DMR appears to be crucial for full paternal *Gnasxl* expression, it is suppression of *Nesp* transcription that is essential for tissue specific paternal repression of *Gnas*. Ectopic paternal expression of *Nesp* results in loss of imprinting of *Gnas* [24]. This occurs because ectopic transcription of *Nesp* results in methylation of the *Exon1A* DMR; the *Exon1A* DMR controls the imprinted expression of *Gnas* [25,28] and methylation of the *Exon1A* DMR results in upregulation of *Gnas* in tissues where *Gnas* shows imprinted expression [4,11,24]. Thus, at the *Gnas* cluster, *Nespas* expression is key to defining paternal gene expression. The primary role of *Nespas* may be to repress *Nesp* expression and induce methylation of the *Nesp* DMR in order that appropriate levels of *Gnas* and *Gnasxl* are maintained on the paternal allele.

## 4. Material and Methods

### 4.1. GenBank Accession Number

The nucleotide numbers referred to in this publication correspond to sequence accession number AL593857.10.

### 4.2. Generation of Targeted Alleles

To generate the *Nespas-T^int2^* allele, a targeting construct was designed to insert a polyadenylation cassette from the rabbit β-globin gene [2] into intron 2 of *Nespas,* between nucleotides 141,635–141,636, in an orientation (designated Ap) that would be expected to truncate *Nespas* (Figure 2a). The construct was generated by homologous recombination in yeast as described previously [4] except that pRAY1 vector [36] was used. The targeting construct was linearised with *Not*I and electroporated into R1 mouse ES cells [37]. Colonies surviving G418 selection were screened by Southern blot analysis of *Afl*II digested DNA probed with genomic fragment probe A (nucleotides 134,606–136,087) that lies 5′ of the targeting construct arm. *Xho*I digested DNA was probed with probe B genomic fragment (nucleotides 147,539–148,139) that lies 3′ of the targeting construct arm (Figure 2a,b). Targeted ES cells were karyotyped, and there was no evidence of chromosomal changes [38]. Chimeras were generated by injecting two correctly targeted ES clones into C57BL/6J blastocysts. Germline transmission of the targeted allele was confirmed by PCR using primers in Table S1. Transmitting male chimeras were crossed with Tg/ACTB-cre/2Mrt/J females that were heterozygous for *β-actin-Cre*, a transgene ubiquitously expressed in embryos at or before the blastocyst stage [39,40]. Offspring were genotyped for the *Nespas-T^int2^* and *β-actin-Cre* alleles and double heterozygotes were crossed with 129S9/SvEvH mice. *Nespas-T^int2^* carriers were maintained on a 129S9/SvEvH background. Excision of the selection cassette was confirmed by PCR amplification across the remaining *loxP* site. The sequence of the primers for detecting the *Nespas-T^int2^* allele and *β-actin-Cre* allele are provided in Table S1. *Nesp^trun^* carriers had previously been generated and were maintained on a C57BL/6 background [19].

The *Nespas-T^int3^* allele was generated by using a targeting construct designed to insert the polyadenylation cassette from the rabbit β-globin gene into intron 4 of *Nespas*, between nucleotides 139,024 and 139,025 and upstream of the *Nesp* promoter in an orientation (designated Ap) that would be expected to truncate *Nespas* (Figure 4a). The construct was generated by homologous recombination in yeast as described above using pRAY-Cre vector [23] that allows self-excision of the selection cassette upon germline transmission. As described above for the generation of the *Nespas-T^int2^* allele, the targeting construct was linearised with *Not*I and electroporated into R1 mouse ES cells [37]. Correctly targeted clones were identified by Southern blot analysis of *Afl*II digested DNA, probed with genomic fragment probe A described above. *Bsr*GI digested DNA was probed with genomic fragment probe B described above (Figure 4a,b). Chimeras were generated by injecting two correctly targeted ES clones into C57BL/6J blastocysts. Germline transmission of the targeted allele was confirmed by PCR using primers in Table S1. *Nespas-T^int3^* carriers were maintained on a 129S9/SvEvH background.

All mouse studies were conducted under guidance issued by the Medical Research Council in “Responsibility in the Use of Animals in Bioscience Research” (May 2008) and under the authority of Home Office Project Licence Number 30/2526 and the approval of the Harwell Ethics Committee. For the characterisation of +/*Nespas-T^int2^* and +/*Nespas-T^int3^*, mice were examined daily and observations recorded using a numerical system on a welfare scoring sheet. From birth onwards, animals were scored for up to 12 parameters affecting feeding, growth, morphology and activity. Mice were housed in Tecniplast IVC 1284L caging with a maximum number of five mice per cage. All cages contained pine bedding (Datesand grade 6) and Datesand rodent tunnels and shredded paper for environmental enrichment. All mice had free access to water and diet (Special diet services (Dietex, Witham, UK) RM3 (E)) in a 12-h light-dark cycle with room temperature 19–22 °C.

### 4.3. Genotyping Assay

PCR quality mouse genomic DNA was prepared from ear/toe clip biopsies by a modified alkaline lysis protocol called HotSHOT [41]. Briefly the biopsies were incubated in 100 µL of 50 mM NaOH for 1 h at 95 °C, then neutralised by adding 10 µL of 1 M Tris-HCl (pH 8) followed by dilution with 100 µL of water. One microlitre of the final volume was added to Reddy Mix PCR Master Mix (Thermo Scientific, Waltham, MA, USA) containing the appropriate primers for PCR amplification.

### 4.4. Mouse Weight Data Analysis

Individual mice were weighed at 14 days. The weight comparisons of mutant mice and their normal sibs were assessed by Student’s *t*-test on the basis of weight ratios within litters as this eliminates weight differences between different litters attributable to varying litter sizes.

### 4.5. Expression Analyses

RNA extraction and reverse transcription-quantitative real-time PCR (RT-qPCR) was performed as described previously [4]. Transcript levels were measured in newborn tissues. *Nesp*, *Nespas* and *Gnasxl* levels were assayed in brain, *Exon1A* in brain and brown fat, and *Gnas* in brown fat. For analysis of *Nespas* in +/*Nespas-T^int2^* and +/*Nespas-T^int3^* Taqman assays for the RT-qPCR were designed 3' and 5' of the insertions with respect to *Nespas* transcription. For +/*Nespas-T^int2^* the 3′ assay was *Nespas* intron 4 (AIGJPKJ; Table S2) and the 5' assay was *Nespas* ex1/ex2 (Mm01248137.m1). For +/*Nespas-T^int3^*, the 3' assay was *Nespas* intron 4 (AI88XP8) [4], the 5' assay for spliced *Nespas* was *Nespas* ex1/ex2 (Mm01248137.m1) and the 5' assay for unspliced *Nespas* was *Nespas* intron 4 (AIGJPKJ). *Nespas* was analysed in +/*Nesp^trun^* using assay *Nespas* ex1/ex2 (Mm01248137.m1). For analysis of *Nesp* in +/*Nespas-T^int2^* and +/*Nesp^trun^* Taqman assay *Nesp* ex1/ex2 (nesp0-N0) was used. *Nesp* was measured in +/*Nespas-T^int−3^* using Taqman assay *Nesp* ex2/ex3 (AIX020D, Table S2, Note: exon 3 of *Nesp* is also exon 2 of *Gnas*). Other assays were for *Gnasxl* (Mm01717466_g1), *Ex1A* (Mm01248152_m1), and *Gnas* (Mm00507037_m1). Samples were analysed in triplicate, and calculations performed using the comparative C_T_ method. The values were normalised to the endogenous reference gene, *Gapdh* (Mm99999915_g1) and the transcript levels were presented as fold change relative to the wild-type sample in relative quantification (RQ) units. Error bars indicate the calculated maximum (RQ_Max_) and minimum (RQ_Min_) expression levels, with a 95% confidence level. Statistical significance was tested using two-sample equal variance, two-tailed distribution Student’s *t*-test and is also represented as non-overlapping error bars if the samples are significantly different (*p* < 0.05).

Poly(A)^+^ RNA for blot analysis was extracted using a FastTrack kit (Invitrogen, Carlsbad, CA, USA). Northerns were carried out as previously described [11].

### 4.6. PCR Product Melting Curve Analysis for Differentiating SNP Based Allelic Expression

Melting curve analysis [42] was performed to determine the parental origin of *Nespas* transcription. Briefly the 90 bp cDNA-derived PCR products, spanning the *Nespas* exon 3 SNP (nucleotide 140,755; C in 129S9/SvEvH and T in SD2) were amplified, in the presence of the double-stranded DNA binding dye LCGreen plus, HotShot Mastermix, and 0.2 mM forward and reverse primers (0.2 mM; Table S2). PCR conditions were: 95 °C for 2 min, then 44 cycles of 95 °C for 30 s, 60 °C for 30 s, 72 °C for 30 s. Following PCR, the products were heated to 95 °C and then rapidly cooled to 15 °C to generate duplexes. The duplexes were melted and the melt curves were obtained using an Idaho Technology LightScanner (Salt Lake City, UT, USA). The decrease in fluorescence was a measure of the bound LCGreen plus being released into solution as the duplex melted. To distinguish allele-specific expression, control cDNA was included to ensure distinct melting curves were obtained for the 129S9/SvEvH and SD2 homoduplexes and heteroduplexes. For the latter, a 1:1 mix of 129S9/SvEvH and SD2 cDNA was used. Each sample was analysed in triplicate.

### 4.7. Methylation Analysis

Methylation at the *Nesp* DMR was analysed by bisulphite analysis. Briefly genomic DNA, extracted from newborn brain using the Allprep kit (Qiagen Ltd. United Kingdom, Manchester, UK), was treated using the EpiTect Bisulfite Kit (Qiagen) and the DNA was amplified using *Nesp* DMR primers [4] then cloned and sequenced. The parental alleles were distinguished using a variant between 129S9/SvEvH and SD2, at nucleotide 140,755, in *Nesp* intron 1. Gel analysis of methylation was performed by digestion of the PCR amplified bisulphite-treated DNA with *Taq*I (recognition sequence TCGA). Digestion products represented methylation of the starting DNA.

### 4.8. Luciferase Reporter Assay to Measure Promoter Activity

Reporter constructs were generated and the assay was done as previously described [25]. The reporter constructs were generated by amplifying the 5′ end of the *Nesp* DMR (nucleotides 139,675–140,530) [14] from mouse PAC clone 583L07 (RPCI21 library) and the product was cloned in both orientations in the *Bgl*II site of the pGL3-Basic vector and pGL3-Enhancer vector (Promega UK, Southampton, UK). The plasmids were transfected into HeLa cells and the pGL3-Control and pGL3-Basic vectors were included as positive and negative controls, respectively. The experiment was done twice whereby for each experiment the construct was tested in duplicate and duplicate readings were taken for each sample.

### 4.9. Transcription Inhibitor Assay

Actinomycin D treatment was performed as previously described [21]. Briefly MEFs were prepared from 13.5 dpc embryos and were used at P14-P18. The cells were cultured for 42 h. At time point 0, the media were removed and the cells were washed with PBS and incubated with media supplemented with 10 µg/mL Actinomycin D (Source BioScience, Nottingham, UK) dissolved in DMSO. Control dishes were incubated with media plus DMSO. At each time point, cells from a control dish and a treated dish were harvested for RNA analysis. Transcript levels were measured by RT-qPCR using Taqman assays *Nespas* ex1/2 for spliced *Nespas* RNA, and intron 4 (AIGJPKJ) for unspliced *Nespas* RNA. The data were taken from three biological replicates, each of which was analysed three times. Each value was normalised to *Gapdh*.

## Supplementary Files

Supplementary File 1

## References

[1] Barlow D.P., Bartolomei M.S. (2014). Genomic imprinting in mammals. Cold Spring Harb. Perspect. Biol..

[2] Sleutels F., Zwart R., Barlow D.P. (2002). The non-coding air rna is required for silencing autosomal imprinted genes. Nature.

[3] Mancini-Dinardo D., Steele S.J., Levorse J.M., Ingram R.S., Tilghman S.M. (2006). Elongation of the kcnq1ot1 transcript is required for genomic imprinting of neighboring genes. Genes Dev..

[4] Williamson C.M., Ball S.T., Dawson C., Mehta S., Beechey C.V., Fray M., Teboul L., Dear T.N., Kelsey G., Peters J. (2011). Uncoupling antisense-mediated silencing and DNA methylation in the imprinted gnas cluster. PLoS Genet..

[5] Meng L., Person R.E., Beaudet A.L. (2012). Ube3a-ats is an atypical rna polymerase ii transcript that represses the paternal expression of ube3a. Hum. Mol. Genet..

[6] Meng L., Person R.E., Huang W., Zhu P.J., Costa-Mattioli M., Beaudet A.L. (2013). Truncation of ube3a-ats unsilences paternal ube3a and ameliorates behavioral defects in the angelman syndrome mouse model. PLoS Genet..

[7] Peters J., Williamson C.M. (2007). Control of imprinting at the gnas cluster. Epigenetics.

[8] Eaton S.A., Williamson C.M., Ball S.T., Beechey C.V., Moir L., Edwards J., Teboul L., Maconochie M., Peters J. (2012). New mutations at the imprinted gnas cluster show gene dosage effects of gsalpha in postnatal growth and implicate xlalphas in bone and fat metabolism but not in suckling. Mol. Cell. Biol..

[9] Fernandez-Rebollo E., Maeda A., Reyes M., Turan S., Frohlich L.F., Plagge A., Kelsey G., Juppner H., Bastepe M. (2012). Loss of xlalphas (extra-large alphas) imprinting results in early postnatal hypoglycemia and lethality in a mouse model of pseudohypoparathyroidism ib. Proc. Natl. Acad. Sci. USA.

[10] Ball S.T., Kelly M.L., Robson J.E., Turner M.D., Harrison J., Jones L., Napper D., Beechey C.V., Hough T., Plagge A. (2013). Gene dosage effects at the imprinted cluster. PLoS ONE.

[11] Williamson C.M., Turner M.D., Ball S.T., Nottingham W.T., Glenister P., Fray M., Tymowska-Lalanne Z., Plagge A., Powles-Glover N., Kelsey G. (2006). Identification of an imprinting control region affecting the expression of all transcripts in the gnas cluster. Nat. Genet..

[12] Williamson C.M., Skinner J.A., Kelsey G., Peters J. (2002). Alternative non-coding splice variants of nespas, an imprinted gene antisense to nesp in the gnas imprinting cluster. Mamm. Genome.

[13] Peters J., Wroe S.F., Wells C.A., Miller H.J., Bodle D., Beechey C.V., Williamson C.M., Kelsey G. (1999). A cluster of oppositely imprinted transcripts at the gnas locus in the distal imprinting region of mouse chromosome 2. Proc. Natl. Acad. Sci. USA.

[14] Kelsey G., Bodle D., Miller H.J., Beechey C.V., Coombes C., Peters J., Williamson C.M. (1999). Identification of imprinted loci by methylation-sensitive representational difference analysis: Application to mouse distal chromosome 2. Genomics.

[15] Liu J., Yu S., Litman D., Chen W., Weinstein L.S. (2000). Identification of a methylation imprint mark within the mouse gnas locus. Mol. Cell. Biol..

[16] Holmes R., Williamson C., Peters J., Denny P., Wells C., Group R.G., Members G.S.L. (2003). A comprehensive transcript map of the mouse gnas imprinted complex. Genome Res..

[17] Robson J.E., Eaton S.A., Underhill P., Williams D., Peters J. (2012). Micrornas 296 and 298 are imprinted and part of the gnas/gnas cluster and mir-296 targets ikbke and tmed9. Rna.

[18] Huang R., Barlow D.P. (2011). Personal Communication.

[19] Chotalia M., Smallwood S.A., Ruf N., Dawson C., Lucifero D., Frontera M., James K., Dean W., Kelsey G. (2009). Transcription is required for establishment of germline methylation marks at imprinted genes. Genes Dev..

[20] Wroe S.F., Kelsey G., Skinner J.A., Bodle D., Ball S.T., Beechey C.V., Peters J., Williamson C.M. (2000). An imprinted transcript, antisense to nesp, adds complexity to the cluster of imprinted genes at the mouse gnas locus. Proc. Natl. Acad. Sci. USA.

[21] Seidl C.I., Stricker S.H., Barlow D.P. (2006). The imprinted air ncrna is an atypical rnapii transcript that evades splicing and escapes nuclear export. EMBO J..

[22] Cattanach B.M., Peters J., Ball S., Rasberry C. (2000). Two imprinted gene mutations: Three phenotypes. Hum. Mol. Genet..

[23] Plagge A., Gordon E., Dean W., Boiani R., Cinti S., Peters J., Kelsey G. (2004). The imprinted signaling protein xl alpha s is required for postnatal adaptation to feeding. Nat. Genet..

[24] Mehta S., Williamson C.M., Ball S., Tibbit C., Beechey C., Fray M., Peters J. (2015). Transcription driven somatic DNA methylation within the imprinted gnas cluster. PLoS ONE.

[25] Williamson C.M., Ball S.T., Nottingham W.T., Skinner J.A., Plagge A., Turner M.D., Powles N., Hough T., Papworth D., Fraser W.D. (2004). A cis-acting control region is required exclusively for the tissue-specific imprinting of gnas. Nat. Genet..

[26] Yu S., Yu D., Lee E., Eckhaus M., Lee R., Corria Z., Accili D., Westphal H., Weinstein L.S. (1998). Variable and tissue-specific hormone resistance in heterotrimeric gs protein alpha-subunit (gsalpha) knockout mice is due to tissue-specific imprinting of the gsalpha gene. Proc. Natl. Acad. Sci. USA.

[27] Stricker S.H., Steenpass L., Pauler F.M., Santoro F., Latos P.A., Huang R., Koerner M.V., Sloane M.A., Warczok K.E., Barlow D.P. (2008). Silencing and transcriptional properties of the imprinted airn ncrna are independent of the endogenous promoter. EMBO J..

[28] Liu J., Chen M., Deng C., Bourc'his D., Nealon J.G., Erlichman B., Bestor T.H., Weinstein L.S. (2005). Identification of the control region for tissue-specific imprinting of the stimulatory g protein alpha-subunit. Proc. Natl. Acad. Sci. USA.

[29] Mehta S. (2012). Imprinting at the Mouse Gnas Cluster.

[30] Ooi S.K., Qiu C., Bernstein E., Li K., Jia D., Yang Z., Erdjument-Bromage H., Tempst P., Lin S.P., Allis C.D. (2007). Dnmt3l connects unmethylated lysine 4 of histone h3 to de novo methylation of DNA. Nature.

[31] Zhang Y., Jurkowska R., Soeroes S., Rajavelu A., Dhayalan A., Bock I., Rathert P., Brandt O., Reinhardt R., Fischle W. (2010). Chromatin methylation activity of dnmt3a and dnmt3a/3l is guided by interaction of the add domain with the histone h3 tail. Nucleic Acids Res..

[32] Dhayalan A., Rajavelu A., Rathert P., Tamas R., Jurkowska R.Z., Ragozin S., Jeltsch A. (2010). The dnmt3a pwwp domain reads histone 3 lysine 36 trimethylation and guides DNA methylation. J. Biol. Chem..

[33] Latos P.A., Pauler F.M., Koerner M.V., Senergin H.B., Hudson Q.J., Stocsits R.R., Allhoff W., Stricker S.H., Klement R.M., Warczok K.E. (2012). Airn transcriptional overlap, but not its lncrna products, induces imprinted igf2r silencing. Science.

[34] Zhao J., Ohsumi T.K., Kung J.T., Ogawa Y., Grau D.J., Sarma K., Song J.J., Kingston R.E., Borowsky M., Lee J.T. (2010). Genome-wide identification of polycomb-associated rnas by rip-seq. Mol. Cell.

[35] Sun B.K., Deaton A.M., Lee J.T. (2006). A transient heterochromatic state in xist preempts x inactivation choice without rna stabilization. Mol. Cell.

[36] Storck T., Kruth U., Kolhekar R., Sprengel R., Seeburg P.H. (1996). Rapid construction in yeast of complex targeting vectors for gene manipulation in the mouse. Nucleic Acids Res..

[37] Nagy A., Rossant J., Nagy R., Abramow-Newerly W., Roder J.C. (1993). Derivation of completely cell culture-derived mice from early-passage embryonic stem cells. Proc. Natl. Acad. Sci. USA.

[38] Evans E.P., Beechey C.V. (2010). Personal Communication.

[39] Lewandoski M., Meyers E.N., Martin G.R. (1997). Analysis of fgf8 gene function in vertebrate development. Cold Spring Harb. Symp. Quant. Biol..

[40] Lewandoski M., Martin G.R. (1997). Cre-mediated chromosome loss in mice. Nat. Genet..

[41] Truett G.E., Heeger P., Mynatt R.L., Truett A.A., Walker J.A., Warman M.L. (2000). Preparation of pcr-quality mouse genomic DNA with hot sodium hydroxide and tris (hotshot). BioTechniques.

[42] Shimomura K., Galvanovskis J., Goldsworthy M., Hugill A., Kaizak S., Lee A., Meadows N., Quwailid M.M., Rydstrom J., Teboul L. (2009). Insulin secretion from beta-cells is affected by deletion of nicotinamide nucleotide transhydrogenase. Methods Enzymol..

