# Supplementary material for: Antisense Activity across the Nesp Promoter is Required for Nespas-Mediated Silencing in the Imprinted Gnas Cluster
Source: Noncoding RNA. 2015 Nov 30;1(3):246–66. doi: 10.3390/ncrna1030246 (PMC5932550; doi:10.3390/ncrna1030246)
Supplement: Supplementary File 1 [file ncrna-01-00246-s001.pdf]

Antisense Activity across the *Nesp* Promoter is Required for *Nespas*-Mediated Silencing in the Imprinted *Gnas* Cluster

Charlotte J. Tibbit, Christine M. Williamson, Stuti Mehta, Simon T. Ball, Mita Chotalia, Wade T. Nottingham, Sally A. Eaton, Deen Quwailid, Lydia Teboul, Gavin Kelsey and Jo Peters

Supplementary Information

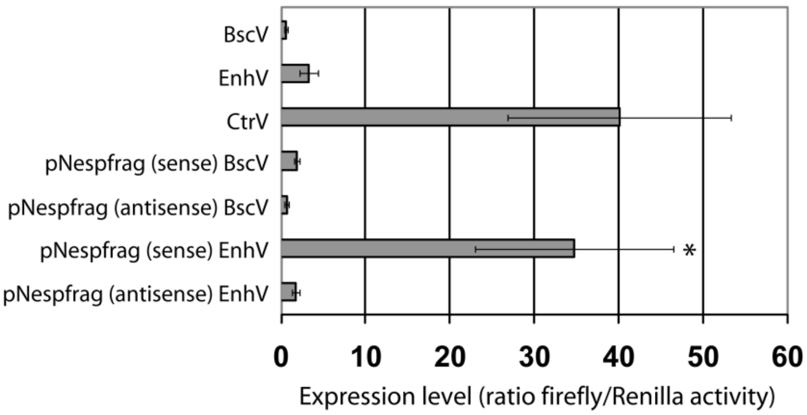

Figure S1. *Nesp* promoter activity detected in HeLa cells using a luciferase reporter assay. Promoter activity was detected from the fragment (nucleotides 139,675–140,530) in the sense orientation and not in the antisense orientation in the Enhancer vector EnhV (designated pNespfrag (sense) EnhV and pNespfrag (antisense) EnhV, respectively). Promoter activity was not detected in the sense and antisense orientation in the Basic vector BscV (designated pNespfrag (sense) BscV and pNespfrag (antisense) BscV, respectively). Thus the *Nesp* promoter lies within nucleotides 139,675-140,530 and an enhancer is required for *Nesp* promoter activity in HeLa cells. BscV, pGL3-Basic Vector (Promega); EnhV, pGL3-Enhancer Vector (Promega); CtrV, pGL3-Control Vector (Promega). Results are mean ± s.e.m of 8 readings. * $P = 1.82 \times 10^{-2}$ *vs.* EnhV by Student's *t*-test (2- tailed).

Table S1. Primer information.

Procedure	Allele	Primer Pair	Forward Sequence (5′-3′)	Reverse Sequence (5′-3′)
Genotyping	*Nespas-T*^{int2}	Alt3′Cre2ndf/CJOR1	TACGTCCAGCCAAGCTAGCTT	CGAATTCAATGTTGCGCACCG
	Nespas-T^{int2} (shows cassette removal)	RabTr9r/NASR3	GAGGAGACAATGGTTGTCAACA	GGCGCAGCAGTCTCAGATCG
	β-actin-Cre	Cre1/Cre2	ATCCGAAAAGAAAACGTTGA	ATCCAGGTTACGGATATAGT
	Nespas-T^{int3}	3′Cre2ndf/R3′armR	AGTACCCCGGGTTCGAAATC	GGAAGATCTATGGACTCAGGTAGGTCCAG
	multiplex assay for detecting either *Nespas-T*^{int2} or *Nespas-T*^{int3}	SE03/SE04 (specific for polyA cassette)	CTGTCTCATCATTTTGGCAAAG	CTCAAGGGGCTTCATGATGT
		SE49/SE50 (amplification control in *Nesp* exon 2)	AGGAGGTTGAGCCTGAGCTG	GCTGCCTGTTTTCCTCCTTG

Table S2. Gene Expression Primers.

Procedure	Transcript	Assay ID	Forward Sequence (5′-3′)	Reverse Sequence (5′-3′)	Probe
RT-qPCR	*Nespas* intron 4	AIGJPKJ	AAAGCATAACACTAGCAGAGTTGTG	CAACAACTAAAACACCCTGCCA	TAGGGATGCATGAAGTCT
	Nesp ex2/*Gnas* ex2	AIX020D	CCGTCCAGATTCTCCTTGTTTTCAT	GATCCTCATCTGCTTCACAATGGT	CTCCAGCACCTTTATCC
Melting curve analysis	*Nespas* exon3		GACTCACCCTCTGGCTC	CCCAGCTTCTCTCCTCAT	
